# Assessment of dual practice among physicians in Cambodia

**DOI:** 10.1186/s12960-020-0461-6

**Published:** 2020-03-12

**Authors:** Keovathanak Khim, Laura N. Goldman, Kristin Shaw, Jeffrey F. Markuns, Vonthanak Saphonn

**Affiliations:** 1 University of Health Sciences (UHS), 73, Monivong Blvd., Khan Daun Penh, Phnom Penh, Cambodia; 2 Boston University, 85 East Newton St., Office 1020, Boston, MA 02118 USA

**Keywords:** Dual practice, Multiple job holding, Cambodia, Health profession regulation, Employment practices, Human resources for health planning

## Abstract

**Background:**

Dual practice and multiple job holding are widespread among health workers throughout the world. Although dual practice can help the financially strained public sector retain skilled workers, there are also potential negative consequences if it is not regulated. In Cambodia, there is substantial anecdotal evidence of dual practice among physicians but there is very little data on the extent and prevalence of the practice. This study was conducted by the University of Health Sciences (UHS) to gain insight in to the employment practices of UHS alumni. Results from this survey may help to inform policymakers in rational planning for future health system development related to capacity building and regulation of human resources for health.

**Methods:**

Data were collected from a self-administered survey of UHS graduates who graduated between 1999 and 2012. A total of 162 medical graduates were randomly sampled from a total of 1867 medical graduates between 1999 and 2012. Contacted individuals were asked to complete a written structured questionnaire regarding demographic characteristics, current employment and types of employment, compensation, and job satisfaction. The response rate of graduates sampled was 49% (79 completed questionnaires). The low response rate was primarily due to the difficulty in locating individuals.

**Results:**

Of 79 respondents, 96% were currently employed at the time of the survey. However, only 63 of the respondents (80%) were working in the healthcare sector. The 16 respondents (20%) not working in healthcare were excluded from further analyses since they are not relevant to dual practice analysis. The vast majority (87%) of respondents are public sector employees (61.9% in public sector only and 25.4% in both public and private sector). 12.7% of respondents only work in the private sector. Almost half (47.6%) of respondents hold more than one job. For income satisfaction, physicians employed in both sectors have higher satisfaction than physicians employed in the public sector only.

**Conclusions:**

As policymakers in Cambodia consider new approaches to regulation of the practice, it is important to know the context of the practice, the benefits to the healthcare system, and the costs. Recognizing the high prevalence of multiple job holding in Cambodia, as evidenced in our survey of UHS medical graduates, contributes to the discussion as important information that can be used toward meaningful reform.

## Background

Dual practice (whereby health workers hold jobs in both the public sector and private sector) and multiple job holding (whereby health workers hold more than one job) are widespread throughout the world [1, 2]. In low- and middle-income countries (LMIC), as many as 87% of healthcare workers supplement their incomes through second jobs, adding 50–80% more to their salaries [3]. Reasons for public sector health professionals engaging in dual practice are many. Besides making extra income, dual practice allows health workers to retain and/or improve clinical skills and to contribute to expanding access to health services. Dual practice may help the financially strained public sector retain skilled workers and support functioning of public services. Allowing heath workers to engage in dual practice also helps sustain the public system where human resources are scarce [4, 5].

Literature indicates that health workers engaging dual practice tend to be more satisfied with their jobs and incomes, despite the need to work longer hours and in poor working environments [6, 7]. The satisfaction may be derived from their ability to see more clients fulfilling their desire for clinical practices and to contribute to people’s health [7, 8]. The satisfaction could be strong enough for them to stay in the public sector job despite many challenges.

There are also potential negative consequences if dual practice is not regulated. These drawbacks include physician absenteeism, pilfering of supplies, informal payments from patients, poor quality of care to the public, and diversion of self-paying patients to a physician’s own private clinic [9, 10].

Although there is a range of potential interventions to manage dual practice [9, 11], there have been no rigorous studies to measure their effectiveness. Many countries, such as Cambodia, have recognized the impact of dual practice and have implemented regulatory measures as part of performance management [6, 12]. For example, the early version of performance-based contracting schemes engaged in 2009 and 2010, the so-called “golden rules,” discouraged private practice [13, 14]. Charity hospitals well-known in Cambodia for providing free medical treatments, such as Kuntha Bopha employ these rules, and their staff are strictly prohibited from private practices.

There is substantial anecdotal evidence of dual practice among health professionals including doctors in Cambodia, but there is very little data on the extent and prevalence of the practice, partly because of the sensitivity of the issue. A brief background on Cambodia may help to explain why dual practice is widespread in Cambodia. A developing country in Southeast Asia, with a population approximately 16 million, Cambodia went through over two decades of civil wars, social unrest, and economic hardship from the late 1960s to the early 1990s. Almost all health professionals perished during the years of the Khmer Rouge between 1975 and 1978, and the country health system, infrastructure, and institutions were completely destroyed [15]. In 1980, there were approximately 20 medical doctors remaining, and the country had to begin rebuilding the healthcare system with extremely scarce resources [16]. Training programs of variable quality for health professionals started hastily in the 1980s to urgently staff health facilities [17]. Some health professionals were trained in short courses along the Cambodian-Thai borders and repatriated to the country after the resolution of the conflict with the United Nations-sponsored democratic election in 1993 [18]. Improving human resources for health was constrained by lack of resources, policy direction, and stewardship. Competing multiple donor agendas, lack of coordination of programs, and little reliable outcome data have contributed to persistent problems with improvements in the healthcare workforce [19].

University of Health Sciences (UHS) is the principle public university in Cambodia training health professionals, including medical doctors. Originally established in 1946, it was closed during the Khmer Rouge years (1975–1979) and reopened in 1980. UHS has subsequently trained the majority of physicians practicing in Cambodia today, with over 4000 medical graduates by 2012 [19]. Private universities, such as International University, started training health professionals in the 1990s. Expansion of training programs for health professionals was part of the package for health sector reform starting in 1995, as part of a strategy to move health services closer to people and communities [20, 21]. This reform increased the number of healthcare facilities according to the size of the population in a coverage area—one primary healthcare facility per 10,000 people and one referral hospital per 100,000 people [22].

During the next two decades, the country’s healthcare system was barely functional. With a small budget and minimal regulation, Cambodia had to rely on international assistance [16]. At the same time, Cambodia implemented a market economy and allowed private sector growth. Inadequate pay, scarcity of public sector jobs, lack of regulation, and new private sector opportunities all contributed to healthcare professionals seeking ways to make ends meet, and to gain clinical experience while at the same time continuing to work in the public sector [3, 10, 23]. The Ministry of Health’s efforts to address poor public health services, poor job performance of health workers, and low retention of rural staff culminated in piloting a number of health financing schemes embedded in health reforms through the subsequent years [24]. There were trials of contracting-out and contracting-in between 1997 and 2002 [25], trial of hybrid contracting between 2004 and 2008 [14, 26], and trial and scale-up of internal contracting from 2009 to the present [27, 28]. Payment subsidies were added to encourage poor people to use public health services, and provided as incentives to health professionals to serve in the public sector [29, 30]. These different forms of performance contracting models included contractual arrangements to address dual practice to varying extents. For example, the “golden rules” attempted to enforce working hours and prevent public sector providers from engaging in private practices during working hours [31, 32].

As Cambodia is currently rebuilding its health system with extremely scarce resources and weak institutions, performance management systems remain barely functional and civil servant codes are rarely enforced [13]. As a result, it is not possible for the Ministry of Health to prevent doctors from engaging in private practice. Some available statistics suggest that in 2016, there were an estimated 6550 physicians working in Cambodia [33]. The Ministry of Health estimated 4500 physicians (68%) run and/or work for private practices based on the number of consultation cabinets, clinics, polyclinics, and private hospitals [34]. However, the 4979 physicians (76%) registered with Medical Council of Cambodia (MCC) in 2016 were predominately from the public sector, indicating many physicians must be employed in both the public and private sector.

Understanding the extent of dual practice in Cambodia is a critical step in developing initiatives and policies to regulate dual practice in line with the current 5-year national strategy for regulation of health professions. In 2014, at the request of the Cambodian Ministry of Health, an in-depth assessment of the Cambodian regulation of health professions was conducted by USAID’s Applying Science to Strengthen and Improve Systems (ASSIST) Project as a first step in strengthening health professions regulation. The assessment reviewed the current regulatory system and conducted interviews with stakeholders from the Ministry of Health, the five health professional councils, and five international development partners. The study found that the current system for health professions regulation was no longer “fit for purpose” to address the current needs and circumstances of Cambodia’s health system, and specifically the system did not adequately deal with the regulation of dual practice among health professionals [35]. The findings resulted in the development of the Health Professional Councils’ National Strategic Plan 2015-2020 [33]. Implementation of this strategic plan has focused on building organizational capacity of the five health professional councils, reviewing legislation, and expanding health professionals’ registration in preparation for the introduction of mandatory registration and licensure. As a result, the percentage of physicians registered with the MCC increased from 41.6% in 2014 to 76% in 2016 [33].

This study was implemented to gain insight into the employment practices of UHS medical alumni. Until this survey, no data had been collected on the practice setting and job holding of UHS graduates, or their satisfaction with job and income. Results from this survey may help to inform policymakers in relation to planning of medical training programs, employment of medical graduates, and regulation of medical doctors.

## Methods

Data were collected from a self-administered survey of UHS medical graduates who graduated between 1999 and 2012. Self-administered surveys were appropriate and feasible with medical doctors because they are literate, extremely busy during working hours, and require flexibility for the time needed to complete questionnaires. This method has been used elsewhere to assess the quality of training and training program improvements [36, 37]. In selecting a sample of medical graduates, a simple random sampling technique was used. A sampling frame of graduates was developed in consultation with the UHS registrar’s office and medical administrative office. There was a total of 1867 medical graduates between 1999 and 2012. Each individual graduate was assigned an ordinal numeric code, and a sample of 162 individual graduates was selected based on random sample numbers generated by the computer.

Upon random selection, the investigators attempted to obtain contact information for the 162 selected individuals. The investigators consulted health-related associations (the Medical Council of Cambodia), the UHS registrar office, and the UHS Information Technology unit to collect contact information for the individuals. However, contact information on paper forms for many selected individuals was not available at these offices, and those available were often outdated. Investigators also contacted the head of the individual’s graduating class if the individual’s contact information was not found. In some cases, Facebook was also utilized to obtain contact information. Those whose contact information was not available were substituted with randomly selected individuals. However, the same problem occurred with most of the substitutes.

The sample size was calculated using the formula for a cross-sectional one group using guidance provided in Aday and Cornelius [38]. The indicator of interest was the proportion of graduates who report having a job within 1 year of graduation. The formula below produced a sample size of 162, split equal between graduate cohorts 1999–2009 and 2010–2012.

*N* = [(*z*_1 − *α*/2_)^2^*P*(1 − *P*)] / *d*^2^, where *N* is the desired sample size; *z*_1 − α_ is the standard errors associated with the confidence intervals, in this case, set at 95%; *P* is the proportion of graduates who report having a job within 1 year of finishing the program at UHS, in this case, it is estimated that at least 70% of graduates have a job within 1 year of graduation; *d* is the desired precision level set at 10% so the proportion of graduates who reported having a job is plus or minus 10%;

Contacted individuals were asked to complete a written structured questionnaire. The questionnaire captures data related to demographic characteristics, current employment, types of employment, compensation, and job satisfaction. Questions on job and income satisfaction used a 3-point scale considering the small sample of respondents. The questionnaire was field-tested in a pilot using ten purposively sampled graduates who were then not eligible for selection in the final survey. Comments and feedback from the pilot were incorporated to improve the design and formulation of the questionnaire.

Data was collected between June and August 2015 by research assistants from the UHS research unit who were trained in questionnaire administration and collection. Data collectors delivered the questionnaire in person to the selected participants and gave oral instructions for completing the survey. They then returned within 1 week to collect the completed questionnaire and check for completeness and accuracy. Each completed questionnaire was given a unique identifying code to ensure the questionnaire remained anonymous. One database was created to link participant names with the identifying codes and this database is kept by the head of the research unit at UHS.

The survey underwent an ethical review by the National Ethics Committee for Health Research of the Ministry of Health in Cambodia and was exempted by the BU IRB. Participation in the survey was purely voluntary and there were no incentives provided.

Data were analyzed on Stata 11. Descriptive statistics (frequencies and means) were generated relating to demographics, employment, types and sectors of employment, satisfaction with current employment, and levels of income. Both bivariate and multivariate correlations were explored using chi-square tests (Tukey HSD test) and ANOVA tests to analyze the relationships between the variables and comparisons of scores between groups.

## Results

A total of 79 medical graduates (49%) completed questionnaires. Of the 79 respondents, 81% were male and 19% were female. A total of 96% were currently employed at the time of the survey. However, only 63 of the respondents (80%) were working in the healthcare sector. The 16 respondents (20%) not working in healthcare were excluded from further analyses since they are not relevant to dual practice analysis.

The following two tables show a breakdown of the sector of work and number of jobs held by the 63 physicians working in healthcare (Tables 1 and 2). The vast majority, 87% of the respondents, are public sector employees (61.9% in public sector only and 25.4% working in both public and private sector). 12.7% of respondents only work in the private sector. In total, 48% reported holding two or more jobs.

**Table 1:** Sector of employment

	Number	Percent
Public	39	61.9
Private	8	12.7
Both sectors	16	25.4
Total	63	100

**Table 2:** Number of current jobs

	Number	Percent
1 job	33	52.4
2 jobs	25	39.7
3 jobs	5	7.9
Total	63	100

Respondents self-reported their average number of working hours per day, average monthly salary, job satisfaction, and income satisfaction. Across all 63 respondents, the average monthly salary was $767.50 U.S. dollars (USD) and the average number of total reported hours worked per day was 10.3 h. While overall job satisfaction was reasonably positive at 2.3, physicians were generally much less satisfied with their income represented by an average satisfaction score of just 1.3. Results by sector and number of jobs held are shown in Tables 3 and 4.

**Table 3:** Working hours, salary, and satisfaction by sector of employment

Sector	Number	Average # hours/day	Average monthly salary ($)	Average job satisfaction	Average income satisfaction
Public	39	9.55	$750.97	2.329	1.17
Private	8	9.37	$737.50	2.125	1.44
Both Sectors	16	12.5*	$827.14	2.344	1.56**
Total	63	10.30	$767.50	2.31	1.30

*p < 0.05 vs. public only and private only **p < 0.05 vs. public only

**Table 4:** Working hours, salary, and satisfaction by number of jobs held

Number of jobs	Number	Average # hours/day	Average monthly salary ($)	Average job satisfaction	Average income satisfaction
1	33	7.72***	$349.67**	2.21	1.15
2	25	12.64	$1,097.00*	2.36	1.46^
3	5	14.6	$2,200.00	2.67	1.53
Total	63	10.30	$767.50	2.31	1.30

*p < 0.05, **p < 0.01, ***p < 0.001 vs. two or three jobs ^*p* < 0.05 vs. one job

As seen in Table 3, the only significant difference in salary and hours worked is that physicians working in both sectors work more hours per day (12.5 h) than physicians working in the public sector only (9.55 h) or the private sector only (9.37 h).

For income satisfaction, physicians employed in both sectors have significantly higher satisfaction (1.56) than physicians employed in the public sector only (1.17). There was no statistically significant difference in salary or job satisfaction based on sector.

As demonstrated in Table 4, physicians working one job work fewer hours per day than physicians working two or three jobs. There is a significant difference in average monthly salary by number of jobs held, with physician income increasing correspondingly with the number of jobs. With a second job, doctors work about 5 extra hours a day, but at the same time, they can triple their monthly salary ($1097 from $350).

Physicians who have two jobs have a significantly higher level of income satisfaction than physicians who have only one job. While the income satisfaction score was also higher with three jobs, the increase was not statistically significant, possibly due to ceiling effect or as a result of the small number of respondents with three jobs. There was no significant difference in job satisfaction by number of jobs held.

There were no differences between men and women in any of the above categories, although the number of women in our survey was small (15 of 79 respondents).

## Discussion

The medical profession in Cambodia is dominated by men, even though the sex ratio in the health professions overall when nurses and midwives are included is close to parity between men and women [39]. Cited explanations for the lack of female doctors include fewer female applicants for medical training, long duration of training (8 to 10 years or more) which conflicts with Cambodian social norms encouraging women to get married early, and gender bias toward men for well-regarded jobs [40]. Other theoretical biases are gender bias in the selection process or in earlier schooling.

This study was the first attempt to assess of the prevalence of multiple job holding by physician graduates Cambodia. An understanding of the context for this widely held practice is an important step toward consideration of how to develop policies to improve quality and equity in healthcare. The literature on this topic is clear that the personal gain to the doctor through supplementation of low public sector salaries with a second job is a significant driver [41]. Other major factors include the lack of performance management in the public sector [4], possibly viewed as ad hoc permission to engage in dual practice in exchange for maintaining a public sector job [5], and a desire to see more patients and gain clinical skills. Doctors know they are needed in the public system, and quitting the public sector job in times of dire need could imply a stewardship problem [42].

Despite low salaries, public sector jobs remain highly beneficial to doctors in many developing countries. Public sector positions often provide valued government-associated social security benefits [43]. Jobs in public hospitals provide physicians access to patients, training, and facilities that are essential to building private practices. Public sector jobs that are linked to teaching in medical schools and universities bestow prestige through professorships [9, 44]. As a result, in many countries holding a public sector position is critical to building a successful private healthcare business and clinical practice [45]. This is true in Cambodia where public sector jobs are highly valued, giving physicians access to information, opinions of influential doctors, recruitment of patients, privileges for treating and referring patients, and an opportunity to make a contribution to the community [10].

On an institutional level, by offsetting non-competitive salary with access to patients, facilities, training, and teaching opportunities, governments can retain the most highly skilled doctors in public facilities. In the public sector, doctors provide care for the poor, and many also train medical students and junior doctors. Governments are able to lessen the significant cost of adequately training and employing sufficient health workers to deliver on ambitious health quality goals [46].

Globally, the disadvantages of having publicly employed doctors also operate their own private clinics have been widely reported [1]. Low public salaries have been used as justification for poor behavior. Absenteeism, pilfering of supplies, informal payments, poor quality of care to the public, and diversion of self-paying patients to a physician’s own private clinic are common [9, 10]. Public healthcare workers are felt to lack motivation and public perception of the quality of care is low, leading patients to bypass public facilities for private clinics, often at considerable personal cost [10]. As a result, different schemes to regulate the practice have been employed. These vary from complete prohibition (Canada, China, India, Greece) to varying levels of regulation, including restriction of private sector salaries (UK); incentives for exclusive public sector employment including higher salaries (Spain, Portugal, Thailand, Italy, India, Norway); allowing private sector activities in public facilities (Italy, Austria, Germany, France); self-regulation; minimization of the public sector in healthcare service delivery (USA); and extensive regulation of the private sector (Canada). A Cochrane Review found the literature describing these different approaches are only descriptive in nature. Outcomes in relationship to health quality, cost, and equity have not been measured. At the present time, there is no evidence to evaluate the effectiveness of different interventions in the management of dual job holding in healthcare [11].

Dual practice of publicly employed doctors and health professionals contribute to increased availability of private health services in Cambodia, and the majority of patients utilize the private sector [47]. A study by the Cambodian Ministry of Health found that two thirds of first-contact care in the health system occurred in the private sector, and 60% of Cambodian healthcare costs were out-of-pocket [48].

Most of the physicians who responded to our survey (87%) have at least one job in the public sector. Roughly half of the doctors surveyed have multiple jobs. Doctors with multiple jobs have higher salaries and also higher satisfaction with their income. Multiple job holding can be public–private, public–public, or private–private. In Cambodia, many health professionals, including doctors on salary in public hospitals also have private practices [49]. As mentioned above, performance evaluations are poorly managed and the codes of civil servants are rarely enforced [13]. This could be seen as an implicit approval of private practice in exchange for retaining healthcare workers in the public system [5].

Our survey indicates that there is a contrast between job satisfaction and income satisfaction. Doctors working in both the public and private sectors scored above 2 on job satisfaction, suggesting they were satisfied with their jobs. Both groups reported scores less than 2 on income satisfaction. Not surprisingly, doctors working in both sectors have higher income satisfaction than doctors working in the public sector alone. Yet, the score of income satisfaction remains low (less than 2 “somewhat satisfied”), which indicates that the increased income from multiple job holding does not necessarily result in increased satisfaction with the work. This suggests that while income may contribute to increased job satisfaction, it is not the only driver and improving other conditions such as workload and organizational environment may be a more feasible approach to increase job satisfaction [50].

In our study 38% of sampled physicians work in the private sector (12.7% private sector only and 25.4% in both the private and public sectors). Of the physicians working in the public sector, 29% reported also working in the private sector. This likely under-represents physicians working in the private sector. This may be due to sampling bias, as we were not able to contact half the doctors in the survey, or reporting bias in that many of our respondents may be reluctant to admit to working in a private practice. It is also possible, however, that in Cambodia fewer public sector physicians work in the private sector than is reported in the literature elsewhere. The relatively low number is consistent with the fact that the number of physicians that register with the Medical Council as private sector doctors is lower than expected [33].

### Limitations

This study has a number of limitations. The sample size was small, and the response rate was low. The sample was biased to individuals we were able to contact in person to complete the survey. Under-reporting of income was likely. In the context of low pay for public sector workers, many doctors may be reluctant to report high incomes due to social norms. Our survey did not ask respondents to specify which sector each individual job belonged to, so our analysis was not able to determine private–public job combinations. We did not collect any qualitative data which could have been useful in adding understanding about dual practices and satisfaction with job and income.

## Conclusions

Recognizing the high prevalence of multiple job holding in Cambodia, as evidenced in our survey of UHS medical graduates, can contribute to the debate by policymakers on possible schemes that can be developed toward meaningful health system reform. Regulation of dual practice of medical doctors needs to be considered within the overall framework of the healthcare system, country context, and norms. Regulators must take into account income satisfaction of doctors and the feasibility of other institutional governance and performance management mechanisms.

## Data Availability

The datasets used during the current study are available from the corresponding author on reasonable request.

## Abbreviations

ASSIST: Applying Science to Strengthen and Improve Systems
LMIC: Low- and middle-income countries
MCC: Medical Council of Cambodia
MOH: Ministry of Health
UHS: University of Health Sciences
BU: Boston University

## References

[1] Jan S (2005). *Dual job holding by public sector health professionals in highly resource-constrained settings: problem or solution?*. Bull World Health Organ.

[2] Ferrinho P (2004). *Dual practice in the health sector: review of the evidence*. Hum Resour Health.

[3] Jan, S., et al., *Dual job holding by public sector health professionals in highly resource-constrained settings: problem or solution?* Bulletin of the World Health Organisation, 2005. 83(10). PMC2626421 16283054

[4] ENG, N. and D. CRAIG, *Could a decentralised human resource management system in Cambodia strengthen performance and accountability?* , in *The many faces of public management reform in the Asia-Pacific region: Research in public policy analysis and management*. 2009, Emerald Group Publishing Limited.

[5] Pak, K., et al., *A critical literature review on accountability and neo-patrimonialism in Cambodia*. 2007, Cambodia Development Resources Institute (CDRI): Phnom Penh, Cambodia.

[6] Khim K (2016). Are health workers motivated by income?. Job motivation of Cambodian primary health workers implementing performance-based financing. Glob Health Action.

[7] Halcomb E, Smyth E, McInnes S (2018). *Job satisfaction and career intentions of registered nurses in primary health care: an integrative review*. BMC Fam Pract.

[8] Lu H (2012). *Job satisfaction among hospital nurses revisited: a systematic review*. Int J Nurs Stud.

[9] Gonzalez C, Cuadrado C (2019). *Interventions to reduce the impact of dual practice in the public health sector*. Medwave.

[10] Ferrinho, P., et al., *Dual practice in the health sector: review of the evidence.* Hum Resour Health, 2004. 2(14). 10.1186/1478-4491-2-14 PMC529467 15509305

[11] Suzanne N. Kiwanuka et al., *Interventions to Manage Dual Practice among Health Workers.* The Cochrane Database of Systematic Reviews, 2011. No. 7 (July 6, 2011): CD008405, 10.1002/14651858.CD008405.pub2. 10.1002/14651858.CD008405.pub2 PMC6791302 21735429

[12] Houle SK (2012). *Does performance-based remuneration for individual health care practitioners affect patient care?: a systematic review*. Ann Intern Med.

[13] Khim K, Annear LP (2013). *Strengthening district health service management and delivery through internal contracting: lessons from pilot projects in Cambodia*. Social Science & Medicine.

[14] BTC, *Provision of basic health services in Kampong Cham province - preparatory document for streeting committee meeting*. 2009, Belgium Technical Cooperation: Phnom Penh.

[15] GOTTESMAN E (2003). *Cambodia after the Khmer Rouge: Inside the politics of nation building*.

[16] Annear LP (1998). *Health and development in Cambodia*. Asian Studies Review.

[17] Lanjouw S, Macrae J, Zwi AB (1999). *Rehabilitating health services in Cambodia: the challenge of coordination in chronic political emergencies*. Health Policy Plan.

[18] OVESEN, J. and I.-B. TRANKELL, *Cambodians and their doctors: a medical anthropology of colonial and post-colonial Cambodia*. 2010, Copenhagen, Denmark: NIAS Press.

[19] UHS, *University of Health Sciences Strategic Plan 2014-2018: Together Towards Excellence in Health Sciences Training and Research*. 2014, University of Health Sciences: Phnom Penh, Cambodia.

[20] KNOWLES, J., *Health sector reform in Cambodia*. 1996, Abt Associates Inc: Baltimore, MD.

[21] MOH and WHO, *Health in Cambodia: Development Goals and Plans*. 1995, Royal Government of Cambodia: Phnom Penh.

[22] MOH, *Guide for developing operational health districts in Cambodia*. 1996, Health planning and statistics unit, the Ministry of Health: Phnom Penh.

[23] Lerberghe VW (2002). *When staff is underpaid: dealing with the individual coping strategies of health personnel*. Bulletin of the World Health Organization.

[24] Khim, K., P. Ir, and P. Annear, *Factors driving change in the design, implementation and scaling-up of contracting for health service delivery in Cambodia 1997-2015.* Health Systems and Reform, 2017. 3(2). 10.1080/23288604.2017.1291217 31514672

[25] Leovinsohn B, Harding A (2005). *Buying results? Contracting for health service delivery in developing countries*. Lancet.

[26] BTC, *Provision of basic health services in Siem Reap and Odormeanchey province - Preparatory document for steering committee meeting*. 2009, Belgium Technical Cooperation: Phnom Penh.

[27] Khim, K. and L.P. Annear, *The transition to semi-autonomous management of district health services in Cambodia*, in *Improving health sector performance: Institutions, motivation and incentives. An international conference organised by Cambodia Development Resource Institute and Oxford Policy Institute, April 2010*, H. Jalilian and V. Sen, Editors. 2011, Institute for Southeast Asian Studies, Singapore: Phnom Penh.

[28] MOH., *Health Strategic Plans 2008-2015*. 2008, Department of Planning and Health Information, Ministry of Health: Phnom Penh.

[29] Zhu A (2019). *Analysis of strategies to attract and retain rural health workers in Cambodia, China, and Vietnam and context influencing their outcomes*. Human Resources for Health.

[30] Van de Poel E (2014). *Can vouchers deliver? An evaluation of subsidies for maternal health care in Cambodia*. Bull World Health Organ.

[31] MOH, *Special Operating Agency Manual*. 2009, Ministry of Health: Phnom Penh, Cambodia.

[32] MOH., Service Delivery Grants – Operational Manual (2008). Department of Planning and Health Information (DPHI).

[33] Project ASSIST (2016). *USAID ASSIST Project: Cambodia Country Report FY16*.

[34] MOH, *Report on health sector achievements in 2017 and goals for 2018*, DPHI, Editor. 2018, Ministry of Health: Phnom Penh.

[35] Clarke D (2016). *Strengthening health professions regulation in Cambodia: a rapid assessment*. Hum Resour Health.

[36] Copeland HL, Hewson M, Weiker G. *Consumer evaluation of educational programs: using questionnaires completed by alumni.* 1998, The Annual Meeting of the American Educational Research Association: San Diego. CA. .

[37] Curran, D., et al., *An alumni survey as a needs assessment for curriculum improvement in obstetrics and gynecology.* Journal of Graduate Medical Education, 2012. September: p. 317-321. 10.4300/JGME-D-11-00122.1 PMC3444184 23997875

[38] ADAY, L.A. and J.L. CORNELIUS, *Designing and conducting health surveys: A comprehensive guide* 2006, San Francisco, CA: John Wiley & Sons, Inc.

[39] Vong S (2019). *Why are fewer women rising to the top? A life history gender analysis of Cambodia's health workforce*. BMC Health Serv Res.

[40] MOWA, *Neary Rattanak 4: Five-Year Strategic National Plan for Gender Equality and the Empowerment of Women 2014-2018*. 2014, Ministry of Women's Affairs: Phnom Penh, Cambodia.

[41] Grepin KA (2016). *Private sector an important but not dominant provider of key health services in low- and middle-income countries*. Health Aff (Millwood).

[42] KAPLAN DA, et al. *Human resource governance: what does governance mean for the health workforce in low- and middle-income countries?* Hum Resour Health. 2013;11. 10.1186/1478-4491-11-6 PMC3584723 23414237

[43] Garcia-Prado A, Gonzalez P (2007). *Policy and regulatory responses to dual practice in the health sector*. Health Policy.

[44] Gonzalez P, Macho-Stadler I (2013). *A theoretical approach to dual practice regulations in the health sector*. J Health Econ.

[45] Garcia-Prado A, Gonzalez P (2011). *Whom do physicians work for? An analysis of dual practice in the health sector*. J Health Polit Policy Law.

[46] Berman, P. and D. Cuizon, *Multiple public-private jobholding of health care providers in developing countries: an exploration of theory and evidence*. 2004, Health Systems Resource Centre, DFID, http://www.heart-resources.org/wp-content/uploads/2012/10/Multiple-public-private-jobholding-of-healthcare-providers.pdf.

[47] NIS. DGH. ICF International, Cambodia Demographic and Health Survey (2014). 2015, Ministry of Planning.

[48] MOH, Estimating Health Expenditure in Cambodia: National Health Accounts Report (2012–2014 Data). Ministry of Health (MOH): Phnom Penh. Cambodia. 2015.

[49] Rotem, A. and J. Dewdne, *Report for Ministry of Health, Cambodia: Mid-Term Review of Health Workforces Development Plan 2006 - 2011*. 2011, Ministry of Health Phnom Penh, Cambodia.

[50] Mohammed Abdullah Al Maqbali, *Factors that influence nurses’ job satisfaction: a literature review* Nursing Management, 2015. 22(2): p. 30-37. 10.7748/nm.22.2.30.e1297 25921909

